# Deciphering Molecular Mechanism Underlying Hypolipidemic Activity of Echinocystic Acid

**DOI:** 10.1155/2014/823154

**Published:** 2014-02-11

**Authors:** Li Han, Peng Lai, Jun-Rong Du

**Affiliations:** Department of Pharmacology, Key Laboratory of Drug Targeting and Drug Delivery Systems, Ministry of Education, West China School of Pharmacy and Translational Neuroscience Center, Sichuan University, Chengdu 610041, China

## Abstract

Our previous study showed that a triterpene mixture, consisting of echinocystic acid (EA) and oleanolic acid (OA) at a ratio of 4 : 1, dose-dependently ameliorated the hyperlipidemia and atherosclerosis in rabbits fed with high fat/high cholesterol diets. This study was aimed at exploring the mechanisms underlying antihyperlipidemic effect of EA. Molecular docking simulation of EA was performed using Molegro Virtual Docker (version: 4.3.0) to investigate the potential targets related to lipid metabolism. Based on the molecular docking information, isotope labeling method or spectrophotometry was applied to examine the effect of EA on the activity of 3-hydroxy-3-methylglutaryl coenzyme A (HMG-CoA) reductase, acyl-CoA:cholesterol acyltransferase (ACAT), and diacylglycerol acyltransferase (DGAT) in rat liver microsomes. Our results revealed a strong affinity of EA towards ACAT and DGAT in molecular docking analysis, while low binding affinity existed between EA and HMG-CoA reductase as well as between EA and cholesteryl ester transfer protein. Consistent with the results of molecular docking, *in vitro* enzyme activity assays showed that EA inhibited ACAT and DGAT, with IC_50_ values of 103 and 139 **μ**M, respectively, and exhibited no significant effect on HMG-CoA reductase activity. The present findings suggest that EA may exert hypolipidemic effect by inhibiting the activity of ACAT and DGAT.

## 1. Introduction

Hyperlipidemia is a key pathogenic factor for the development of cardiovascular and cerebrovascular diseases, such as atherosclerosis, hypertension, coronary heart disease, and brain stroke [1]. Pharmacotherapy is the primary way of ameliorating hyperlipidemia, among which statins and fibrate derivates are the most commonly used cholesterol-and triglyceride-lowering drugs [2]. However, a substantial number of patients treated with these lipid-lowering drugs fail to effectively improve dyslipidemia [3]. Moreover, several adverse effects such as hepatic dysfunction and muscle injury are reported with statin and fibrate therapy [4, 5]. In the recent years, with the advent of novel treatment targets for hyperlipidemia, numerous researches have been carried out in order to develop effective and safe lipid-lowering agents from natural products and synthetic compounds.

Phytochemical and pharmacological studies have demonstrated that triterpenoidal saponins are the main active constitutes of *G. sinensis* [6]. Our previous study demonstrated that oral administration of a pentacyclic triterpene mixture isolated from* G. sinensis* fruits (6 or 12 mg/kg/day), consisting of echinocystic acid (EA) and oleanolic acid (OA) at a ratio of 4 : 1, effectively improved the hyperlipidemia and subsequent atherosclerosis in rabbits fed with high fat/high cholesterol diets, suggesting the main constitute EA might be responsive to the hypolipidemic effect of triterpene extract from *G. sinensis* fruits *in vivo* [7]. It is reported that OA significantly inhibited the diacylglycerol acyltransferase (DGAT) from rat liver microsomes, lowered plasma cholesterol by inhibiting intestinal acyl-CoA:cholesterol acyltransferase (ACAT) activity in high-fat-fed hamsters, and protected against isoproterenol-induced myocardial ischemia in rats via antihyperlipidemic, antioxidative, and antiarrhythmic properties as well as its membrane-stabilizing action [8–10]. In addition, EA isolated from *G. sinensis* fruits prevented rat acute myocardial ischemia induced by isoproterenol and vasopressin [11]. Collectively, pentacyclic triterpenes, such as OA and EA, show potential therapeutic effects for cardiovascular diseases associated with dyslipidemia. However, the molecular mechanisms underlying the antihyperlipidemic effect of EA largely remain unclear.

A large body of studies has demonstrated that lipid profiles are governed by various enzymes and proteins, such as 3-hydroxy-3-methylglutaryl coenzyme A (HMG-CoA) reductase, cholesteryl ester transfer protein (CETP), ACAT, and DGAT. HMG-CoA reductase acts as the rate-limiting enzyme of cholesterol biosynthesis pathway by catalyzing the conversion of HMG to mevalonate [4]. CETP promotes the transfer of cholesteryl esters from antiatherogenic high-density lipoprotein (HDL) to proatherogenic lipoproteins such as very low-density lipoprotein (VLDL) and low-density lipoprotein (LDL), and CETP inhibition or deficiency can effectively retard atherogenesis by increasing HDL and decreasing LDL [12]. ACAT catalyzes cholesterol esterification from cholesterol and fatty acyl coenzyme A, followed by subsequent cholesterol absorption, whereas ACAT inhibition is a therapeutic strategy for hypercholesterolemia and atherosclerosis through lowering cholesterol levels, diminishing the assembly and secretion of apolipoprotein B-containing lipoproteins such as VLDL, and inhibiting the formation of foam cells in the arterial walls [13]. DGAT catalyzes the formation of triglyceride in the final step of triglyceride biosynthesis via covalently linking a fatty acyl CoA with the free hydroxyl group of diacylglycerol, and DGAT inhibition is beneficial for the treatment of hypertriglyceridemia via decreasing serum triglyceride levels [14, 15]. Therefore, in order to explore the potential mechanisms of antihyperlipidemic effect of EA, the present study first performed the molecular docking of EA with HMG-CoA reductase, CETP, ACAT, and DGAT to predict the potential targets, and further investigated the effects of EA on the possible targets in *in vitro* rat liver microsomes.

## 2. Materials and Methods

### 2.1. Materials

Tris, phosphatidylserine, (R.S)-3-hydroxy-3-methylglutaryl coenzyme A [(R.S)-HMG-CoA], nicotinamide adenine dinucleotide phosphate (NADPH), 1,2-glyceryl dioleate, lecithin, and 3-oleic acid glycerol were purchased from Sigma-Aldrich (St. Louis, MO, USA). [1-^14^C] oleoyl-CoA was purchased from New England Nuclear Corporation (Boston, USA). Scintillation solution was purchased from Lipoluma, Lumac Co (Clanton, USA). BCA protein assay kit was from Boster Biological Technology (Wuhan, China). Pravastatin was obtained from Bristol-Myers Squibb (Shanghai, China). Other reagents were obtained from commercial sources.

### 2.2. Isolation of Echinocystic Acid (EA)

**Figure 1:**
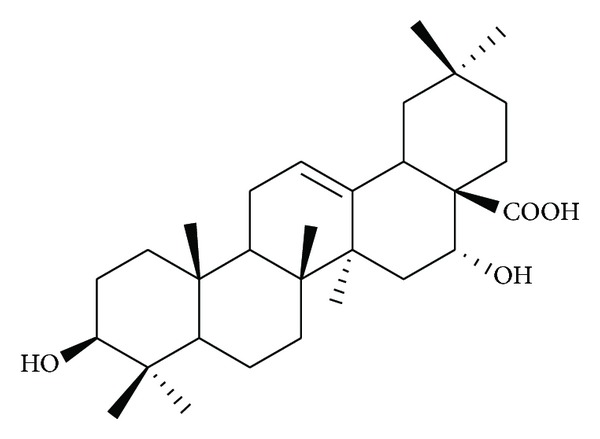
Chemical structure of echinocystic acid (EA).

The fruits of *Gleditsia sinensis* Lam. (*G. sinensis*) were collected from Sichuan province in China, and the aqueous extract was prepared as we described previously [7], followed by isolation of echinocystic acid (EA) using high-performance liquid chromatography (HPLC). In brief, chromatographic separation was conducted on a column (40 × 500 mm) filled with reverse phase C18 silica gel. The mobile phase (MeOH-H_2_O, 8 : 2, *v* : *v*) was conveyed to the column at a flow rate of 10 mL/min and the eluate was detected at 215 nm by diode array detector. EA ((3*β*,16*α*)-3,16-dihydroxyolean-12-en-28-oic acid, Figure 1) was collected and identified by spectral techniques including ^1^H-NMR and ESI-MS and the purity of EA was examined based on the percentage of total peak areas by HPLC. In the present study, EA (purity > 98%) stock solution was prepared via mixing thoroughly 10 *μ*L Tween 80, 20 *μ*L polyethylene glycol 200, and 100 *μ*L water with 1 mg EA and diluted with water prior to enzyme activity assay.

### 2.3. Molecular Docking Analysis

Molecular docking was performed by Molegro Virtual Docker (MVD) 4.3.0 tool adopting MolDock SE algorithm and Rerank scoring function as described previously [16]. The crystal structures of docked receptors (HMG-CoA reductase (PDB ID:1R31), CETP (PDB ID:2OBD), ACAT (PDB ID:1WL4), and DGAT (PDB ID: 1 K30)) were retrieved from RCSB Protein Data bank (http://www.rcsb.org/). The crystal structure of EA as docked ligand was available from NCBI's PCCompound database (http://pubchem.ncbi.nlm.nih.gov/).

Before docking, the receptor protein imported into the MVD software was preprocessed by removing cofactor, crystal water, and initial ligand, and then the free receptor protein was modified by adding the surface which shows the charge distribution of the receptor protein. Following the import of ligand EA into the MVD, molecular docking simulation of EA was performed to investigate the binding affinity of the receptor protein by computing the binding free energy between EA and the possible binding site of the receptor according to the software manual. The possible binding modes between EA and the ideal receptors were then analyzed according to the results of molecular docking. We conducted the docking of EA with HMG-CoA reductase, CETP, ACAT, and DGAT, receptively. The docking was done with the setting of the MVD as follows: (a) score: MolDock score [Grid]; (b) grid resolution (A): 0.30; (c) max iterations: 1500; (d) max population size: 50; (e) other parameters were the default setting. The pictures were prepared using MVD of 4.3.0 version.

### 2.4. Preparation of Rat Liver Microsomes

Male SPF Sprague-Dawley rats, 260~300 g, were purchased from Chongqing Tengxin Animal Center (Chongqing, China). The procedure of animal experiment was carried out following the institutional guidelines of Animal Care and Use Committee at Sichuan University.

Rat liver microsomes were prepared as described previously [17, 18]. In brief, the rats were killed by decapitation. Then, the liver was swiftly removed; rinsed by cold 0.9% NaCl solution; weighed,;cut finely into pieces with scissors; placed into 9 vol. of cold homogenization medium containing 137 mM NaCl, 2.7 mM KCl, 10 mM Na_2_HPO_4_, 2 mM KH_2_PO_4_, 100 mM sucrose, 10 mM EDTA, and 2 mM DTT; and homogenized in a Teflon homogenizer for 10 min. The homogenate was subsequently centrifuged at 12000 ×g for 20 min at 4°C (Beckmann refrigerated centrifuge TJ-6). The supernatant fraction was sucked out and centrifuged again at 100000 ×g for 60 min at 4°C after adding a certain amount of 1 M CaCl_2_ solution (final concentration of CaCl_2_: 8 mM). The precipitation fraction (microsomes) was acquired via the removal of the supernatant fraction. The prepared microsomes were, respectively, resuspended in KCl-Tris-HCl buffer solution (Tris-HCl: 10 mM, KCl: 100 mM, and pH 7.4) of 100–200 *μ*L or 2.5 M sucrose solutions, mixed thoroughly, and then stored in −80°C for assays of microsomal enzyme activities.

### 2.5. Assay of Microsomal HMG-CoA Reductase Activity

Effect of EA on HMG-CoA reductase activity was tested via spectrophotometry using HMG-CoA and cofactor NADPH as described previously [19]. Briefly, the reactive mixture containing 100 *μ*L of 0.2 mM NADPH, 600 *μ*L of phosphate buffer (pH 6.8) (composed of 300 mM KCI, 240 mM potassium phosphate, 6 mM EDTA, and 15 mM DTT), 100 *μ*L of the prepared microsome suspension (10 mg/mL protein), and 10 *μ*L of the test sample EA (200 mM) or the positive control pravastatin (50 mM) was first monitored at 340 nm using ultraviolet spectrophotometer (UNICO) for HMG-CoA-independent oxidation of NADPH. The reaction was then initiated by adding 100 *μ*L of 1 mM (R.S)-HMG-CoA. After 5 min of incubation at 37°C, the supernatant was sucked out and tested at 340 nm by spectrophotometer for HMG-CoA-dependent oxidation of NADPH. One unit of HMG-CoA reductase was defined as the amount of enzyme that catalyzes the oxidation of 1 *μ*mol of NADPH per gram of microsome protein. The protein concentration was measured by the method of BCA using BSA as the standard. The inhibitory effect of EA or pravastatin was calculated as a percentage of HMG-CoA reductase activity of control group, respectively.

### 2.6. Assay of Microsomal ACAT Activity

Effect of EA on ACAT activity was tested by the isotope labeling method as reported previously [20]. In brief, the prepared microsomes were unfrozen and dissolved at 37°C water bath. The reactive mixture, containing 10 *μ*L of microsome suspension (10 mg/mL protein), 20 *μ*L of 0.5 M potassium phosphate buffer (pH 7.4, 10 mM DTT), 10 *μ*L of BSA (180 mg/mL), 2.0 *μ*L of cholesterol in acetone (20 mg/mL), 130 *μ*L of water, and 10 *μ*L of the test sample EA at a concentration range of 0~400 *μ*M, was preincubated for 30 min at 37°C. The reaction was started by adding 10 *μ*L of [1-^14^C] oleoyl-CoA (0.05 *μ*Ci: final concentration 10 *μ*M). After 30 min incubation at 37°C, the reaction was terminated by adding 1.0 mL of *i*-PrOH-*n*-hexane (4 : 1, *v*/*v*) solution. A mixture of 0.6 mL of *n*-hexane and 0.4 mL of 0.1 M potassium phosphate buffer was subsequently added to the reaction mixture and mixed uniformly by vortexing. Standing for 2 min was allowed to separate the reaction mixture into aqueous and organic phases. The upper organic phase containing the radiolabeled cholesteryl ester products was sucked out. The radioactivity in 100 *μ*L of the upper phase was determined using 4 mL of scintillation cocktail (Lipoluma, Lumac Co.) by a LS6000 Beckman Liquid Scintillation Counter (Beckman Inc). Data were presented as counts per minute (CPM) of [1-^14^C] cholesteryl ester products and the readings were normalized to protein concentrations, which were measured by the method of BCA using BSA as the standard. Effect of EA on ACAT activity was calculated as the percentage of inhibition versus control group. Software Origin 7.5 (OriginLab, USA) was used to draw the relation curve of drug concentrations with the inhibition rate, and the 50% inhibitory concentration (IC_50_) was calculated.

### 2.7. Assay of Microsomal DGAT Activity

Effect of EA on DGAT activity was tested by the isotope labeling method as reported previously [8]. In brief, EA at a concentration range of 0~400 *μ*M was incubated 30 min at 37°C with the prepared microsome suspension (10 mg/mL protein), [1-^14^C] oleoyl-CoA (0.05 *μ*Ci: final concentration 3 *μ*M), 3 mM 1,2-glyceryl dioleate, and the incubation buffer that was composed of 200 *μ*M MgCl_2_, 1 mg/mL fatty acid-free BSA, 100 *μ*M lecithin, 100 *μ*M phosphatidylserine, and 5 mM Tris-HCl (pH 8). After 30 min of incubation, the reaction was stopped by adding chloroform-methanol (1 : 1, *v*/*v*) solution, chloroform and acidified sodium chloride solution (containing 17 mM NaCl and 1 mM H_2_SO_4_). The precooled unlabelled glyceryl trioleate was added to the above reaction mixture. The lipids in the mixture were extracted into the organic solvent by centrifugation at 2500 rpm for 10 min. The lower organic phrase containing lipids was recovered, dried under nitrogen, and redissolved in 100 *μ*L of chloroform. The lipids were then separated by silica gel thin-layer chromatography plate in chloroform : diethyl ether : acetic acid (9 : 6 : 4, *v*/*v*/*v*). Triglyceride-specific bands were scraped after being verified by standards with exposure to I_2_ vapor, and the radioactivity was measured by liquid scintillation counting. Data were presented as CPM of [1-^14^C] triglyceride products and the readings were normalized to protein concentrations. Effect of EA on DGAT activity was calculated as the percentage of inhibition versus control group. Software Origin 7.5 was used to draw the relation curve of drug concentrations with the inhibition rate, and the IC_50_ was calculated.

### 2.8. Statistical Analysis

Data are presented as mean ± SD. Statistical analyses were performed with SPSS 16.0 software. The significance of data between the tested groups was determined by one-way ANOVA. The probability level for statistical significance was set at *P* < 0.05.

## 3. Results and Discussion

### 3.1. Molecular Docking

To predict the potential targets of lipid-lowering effects of EA, we performed the molecular docking of EA with HMG-CoA reductase, DGAT, ACAT, and CETP by MVD 4.3.0 tool using Rerank scoring function, respectively. As shown in Table 1, Rerank scores were recorded and used as the index of binding free energy between the ligand and the receptor protein, which is known to be negatively correlated with binding affinity. The docking results showed that EA exhibited a relatively strong binding affinity with ACAT and DGAT as inferred by their negative Rerank scores, −53 and −41, which indicate low binding free energy; the binding affinity between EA and HMG-CoA reductase was found to be very low, so was the binding affinity between EA and CETP, as evidenced by their positive Rerank scores, +4 and +12. These data indicate that EA has a strong binding affinity with ACAT and DGAT. In addition, it is reported that the negative free energy change (Δ*G*) values suggests a spontaneous interaction and correspond to a spontaneous binding process [21]. Therefore, the binding process between EA and HMG-CoA reductase and the binding between EA and CETP were probably not pontaneous, which implied that there was no specific binding ability of EA to HMG-CoA reductase and CETP. These molecular docking results suggest that ACAT and DGAT rather than HMG-CoA reductase and CETP are likely to be the potential targets of lipid-lowering effects of EA.

**Figure 2:**
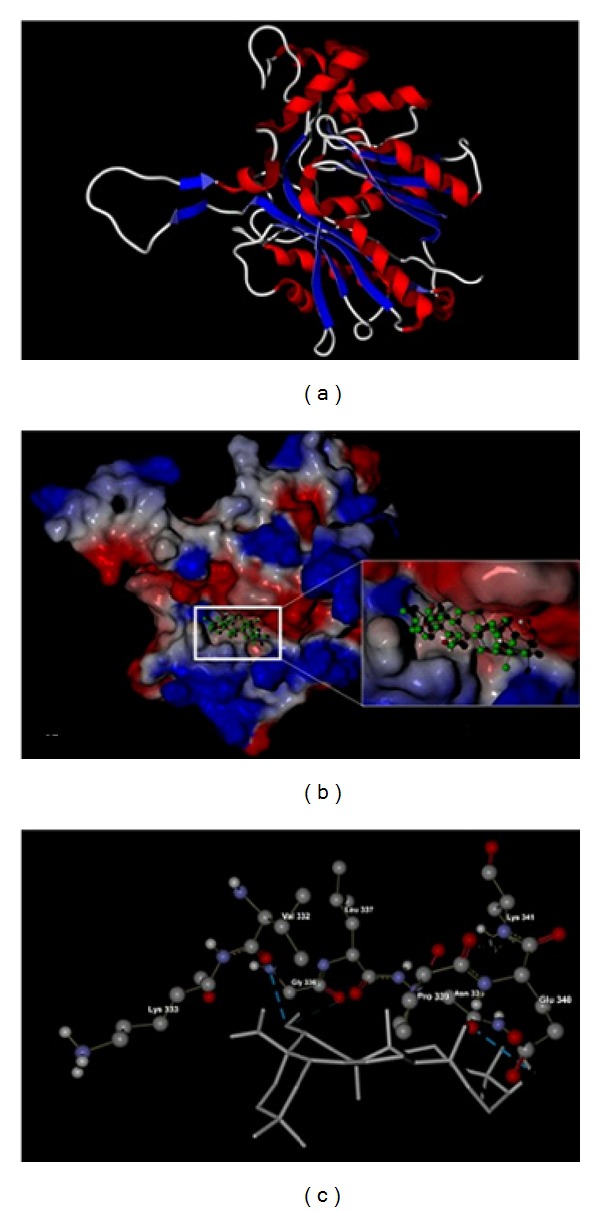
Molecular docking of echinocystic acid (EA) with ACAT in 3D diagram. (a) Three-dimensional structure of ACAT. (b) Optimized docking conformation of EA in the hydrophobic pocket of ACAT. The surface of ACAT was color-coded by electrostatic potential. Red, positive charge; white, neutral; blue, negative charge. (c) Detailed binding mode of EA with ACAT. Dotted blue lines display the hydrogen bonding between the carboxyl group and OH group of EA and amino acid residues Gly 336 and Glu 340 of ACAT.

**Figure 3:**
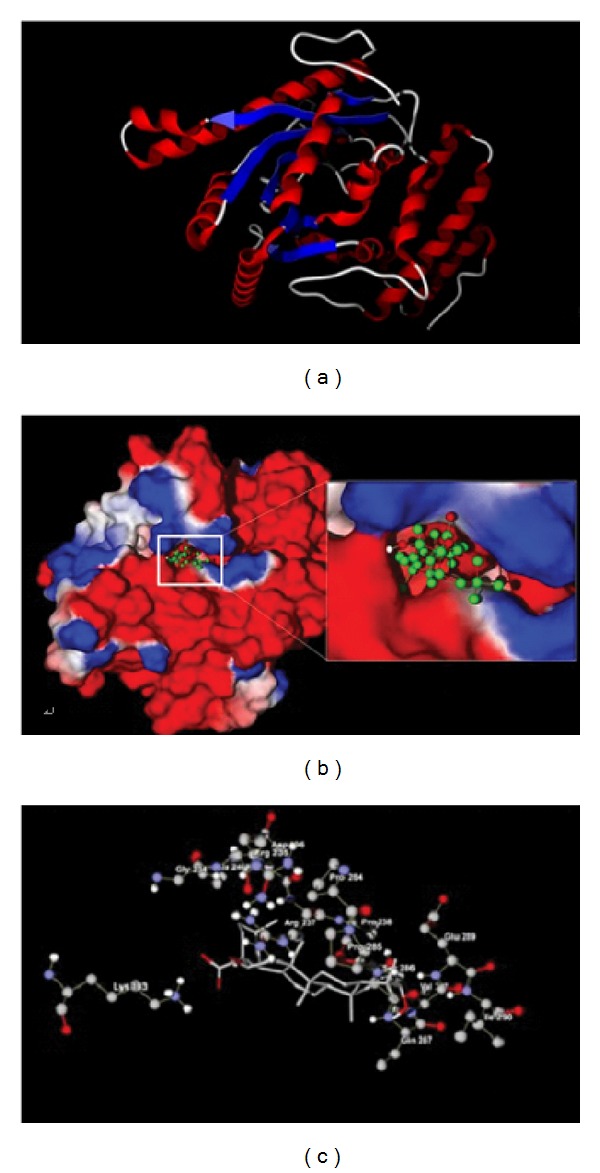
Molecular docking of echinocystic acid (EA) with DGAT in 3D diagram. (a) Three-dimensional structure of DGAT. (b) Optimized docking conformation of EA in the hydrophobic pocket of DGAT. The surface of DGAT was color-coded by electrostatic potential. Red, positive charge; white, neutral; blue, negative charge. (c) Detailed binding mode of EA with DGAT. Dotted blue line displays the hydrogen bonding between the carboxyl group of EA and residue Lys 193 of DGAT.

Following the results mentioned above, we further analyzed the binding modes and interactions of EA with ACAT and DGAT, respectively. The results were shown in Figures 2 and 3. According to the results of MVD docking simulation, EA was most likely bound to ACAT's site within the hydrophobic pocket which is rich in hydrophobic amino acids such as Val, Leu, and Pro, as depicted in Figures 2(b) and 2(c). As a triterpenoid acid, EA has good hydrophobic property, which benefits from binding between the amino acid residues and small molecular compounds with hydrophobic property, so hydrophobic forces may well be one of the main interaction forces behind the binding of EA with ACAT. Furthermore, as seen in Figure 2(c), the OH group and carboxyl group of EA form two hydrogen bonds with residues Gly 336 and Glu 340 of ACAT, respectively. Therefore, the hydrogen bonds may be another interaction force behind the binding of EA and ACAT. As for DGAT, the MVD docking simulation revealed that it had a similar binding mode with EA towards ACAT. EA was also most probably going to bind with DGAT's site within the hydrophobic pocket (Figure 3(b)), which is likely due to the similar structure of DGAT to ACAT since both enzymes belong to the membrane-bound O-acyltransferase (MBOAT) family. However, the pocket, which is wealthy in residue Pro, was obviously not large enough to accommodate the whole structure of EA. It thus apparently decreases the hydrophobic interaction strength between EA and its surrounding residues, which, in turn, may decrease the inhibitory effect of EA on DGAT activity. Moreover, as shown in Figure 3(c), only one hydrogen bond was established between the carboxyl group of EA and residue Lys 193 of DGAT, which may be another reason why the binding affinity of EA with ACAT is stronger than that of EA with DGAT. These results are consistent with that obtained by Rerank scoring function which show that EA has a lower binding free energy and stronger binding affinity with ACAT compared to that with DGAT.

### 3.2. HMG-CoA Reductase Activity

**Figure 4:**
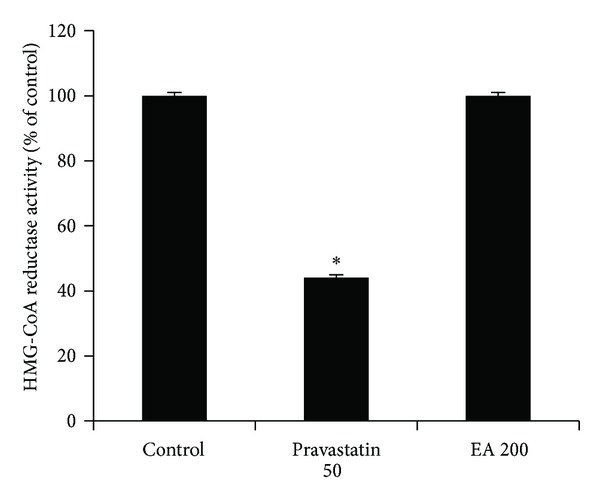
Effect of echinocystic acid (EA) on HMG-CoA reductase activity in rat liver microsomes. Pravastatin was used as a positive control. The inhibitory effect of 50 *μ*M pravastatin (pravastatin 50) or 200**** *μ*M EA (EA 200) was calculated as the percentage of HMG-CoA reductase activity of control group, respectively. Data are expressed as mean ± SD (*n* = 5). *P < 0.05 versus control group, determined by one-way ANOVA.

HMG-CoA reductase is the rate-limiting enzyme of cholesterol biosynthesis pathway and thus is regarded as a major target for regulating hypercholesterolemia. In our previous study, 14-week treatment with a triterpene mixture consisting of 9.6 mg EA and 2.4 mg OA once daily (i.g.) decreased the total cholesterol levels in serum, aorta homogenates, and liver homogenates by 43%, 72%, and 75%, respectively, in hyperlipidemia and atherosclerosis rabbits fed with high fat/high cholesterol diets [7]. By contrast, however, the present molecular docking showed that the binding affinity between EA and HMG-CoA reductase was very low, suggesting that HMG-CoA reductase is not likely to be the potential target of cholesterol-lowering effect of EA. To validate this speculation, we assayed the effect of EA on the activity of HMG-CoA reductase in rat liver microsomes by spectrophotometry. As shown in Figure 4, EA showed no HMG-CoA reductase inhibitory activity even at 200 *μ*M (*P* > 0.05), while pravastatin at a concentration of 50 *μ*M exhibited a significant inhibition (*P* < 0.05) compared with controls. Taken together, the current results demonstrate that the other targets rather than HMG-CoA reductase is responsive to cholesterol-lowering activity of EA *in vivo*.

### 3.3. ACAT Activity

**Figure 5:**
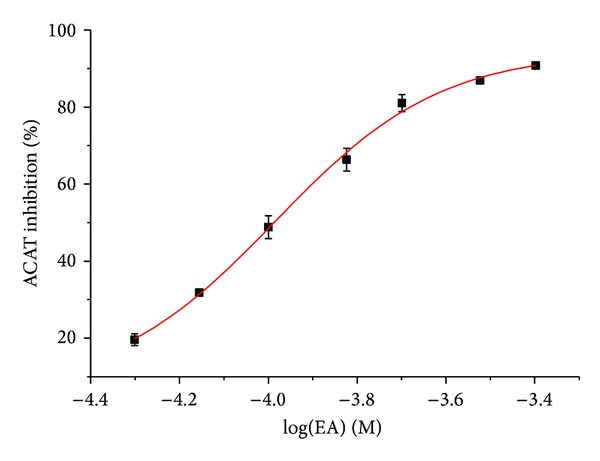
Effect of echinocystic acid (EA) on ACAT activity in rat liver microsomes. The inhibitory effect of EA was calculated as the percentage of inhibition versus control group. Data represent mean ± SD of three independent experiments.

ACAT is regarded as a novel target for the treatment of hypercholesterolemia and atherosclerosis [13]. ACAT inhibitors, such as pactimibe, are reported to have cholesterol-lowering and antiatherosclerotic effects [22]. It is reported that OA at a concentration of 50 *μ*M significantly inhibited ACAT activity in Caco-2 cells, a human intestinal cell line [9]. The present molecular docking showed that the binding affinity between EA and ACAT was much stronger than that between EA and HMG-CoA reductase, suggesting that ACAT is likely to be responsive for cholesterol-lowering effect of EA. Therefore, we investigated the effect of EA on ACAT activity in rat liver microsomes using the isotope labeling method. As shown in Figure 5, EA at a concentration range of 0~400 *μ*M concentration-dependently reduced ACAT activity, with an IC_50_ value of 103 *μ*M, suggesting that ACAT inhibition contributes to the potent cholesterol-lowering effect of EA/OA mixture *in vivo*.

### 3.4. DGAT Activity

**Figure 6:**
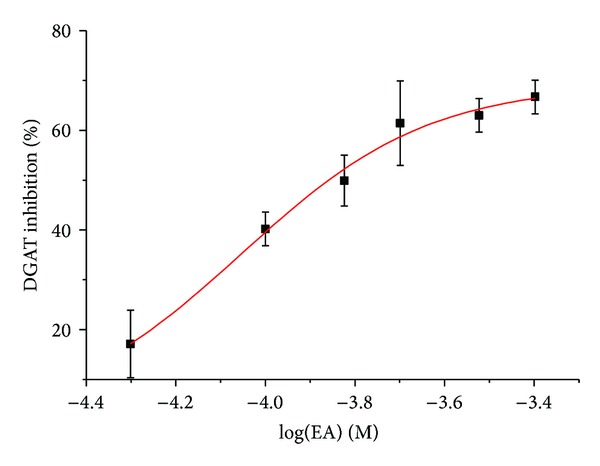
Effect of echinocystic acid (EA) on DGAT activity in rat liver microsomes. The inhibitory effect of EA was calculated as the percentage of inhibition versus control group. Data represent mean ± SD of three independent experiments.

Hypertriglyceridemia is known as a major risk factor of obesity and cardiovascular diseases [23]. DGAT, a key enzyme in triacylglycerol synthesis, is regarded as a potential target for the treatment of triglyceride metabolic disorders [14, 15, 24]. OA is reported to significantly inhibit DGAT from rat liver microsomes [8]. In our previous study, 14-week treatment with a triterpene mixture consisting of 9.6 mg EA and 2.4 mg OA once daily (i.g.) decreased the triacylglycerol levels in serum and aorta homogenates by 54.5% and 29%, respectively, in hyperlipidemia and atherosclerosis rabbits fed with high fat/high cholesterol diets [7]. The molecular docking results showed that EA exhibited a relatively strong binding affinity with DGAT, suggesting that DGAT inhibition is probably associated with triacylglycerol-lowering effect of EA. Therefore, we investigated the effect of EA on DGAT activity in rat liver microsomes using the isotope labeling method. As shown in Figure 6, EA at a concentration range of 0~400 *μ*M concentration-dependently reduced DGAT activity in rat liver microsomes, with an IC_50_ value of 139 *μ*M, suggesting that DGAT inhibition is responsive to triacylglycerol-lowering effect of EA/OA mixture *in vivo*.

Taken together, our current findings show, for the first time, that EA inhibits ACAT and DGAT with IC_50_ values of 103 and 139 *μ*M, respectively, and exhibits no significant effect on the activity of HMG-CoA reductase. These results suggest that EA is a potential natural hypolipidemic agent by inhibiting ACAT and DGAT activity.

## Figures and Tables

**Table 1:** Docking scores of echinocystic acid with the enzymes/protein related to lipid metabolism.

Enzymes	Rerank score
HMG-CoA reductase	+4
CETP	+12
ACAT	−53
DGAT	−41
